# Forty-fourth Annual Commencement of the Medical Department, University of Buffalo

**Published:** 1890-04

**Authors:** 


BUFFALO MEDICAL AND SURGICAL JOURNAL

A MONTHLY REVIEW OF MEDICINE AND SURGERY.

*EDITORS:*

THOS. LOTHROP, M. D. - - - W. W. POTTER, M. D.

All communications, whether of a literary or business character, should be addressed to the editors: 284 FRANKLIN STREET, BUFFALO, N. Y.

### Editorial.

### FORTY-FOURTH ANNUAL COMMENCEMENT OF THE MEDICAL DEPARTMENT, UNIVERSITY OF BUFFALO.

### MEETING OF THE ALUMNI ASSOCIATION.

THE forty-fourth annual commencement of the Medical Department of the University of Buffalo took place Tuesday, March 25, 1890.

At the morning session of the Alumni Association, in the absence of the President, Dr. Shurley, of Detroit, Dr. M. B. Folwell called the meeting to order, and introduced Prof. Parmenter, who gave a brief address of welcome, in which he spoke of the recent changes in the Faculty, recommended a closer connection between the Alumni Association and the Faculty, and exhorted the members of the Association to take a live interest in it and make it a power in the medical profession.

The following officers of the Association were elected for the ensuing year:

President, Peter M. Wise, M. D., Ogdensburg, N. Y.
First Vice-President, M. B. Folwell, M. D., Buffalo, N. Y.
Second " " E. R. Hopkins, M. D., Silver Creek, N. Y.
Third " " W. C. Callanan, M. D., Buffalo, N. Y.
Fourth " " A. G. Ellenwood, M. D.
Fifth " " F. F. Hoyer, M. D., Tonawanda, N. Y.
Permanent Secretary, John J. Walsh, M. D., Buffalo, N. Y.
Recording Secretary, Eugene Smith, M. D., Buffalo, N. Y.
Treasurer, E. C. W. O'Brien, M. D., Buffalo, N. Y.

At the afternoon session Vice-President Dr. Peter M. Wise, now of Ogdensburg, N. Y., presided in place of the president, Dr. Shurley, of Detroit.

Prof. John Parmenter read a paper on The Local Treatment of Erysipelas.

Prof. Chas. G. Stockton presented a paper entitled Clinical Notes on Gastric Faradization.

Prof. Ernest Wende read a paper on The Modern Treatment of Scabies.

The Commencement exercises took place at Music Hall in the evening. A large audience was present to see the conferring of the degree of Doctor of Medicine upon the candidates for this title.

In the absence of the Chancellor, the Vice-Chancellor, Hon. James O. Putnam, awarded diplomas to the following class :

Stanley C. Babcock, Buffalo ; Will H. Baker, Wilhelm ; Nathan E. Beardsley, Pine Valley ; Ida C. Bender, Buffalo ; Harry S. Benham, Honeoye Falls ; George M. Brockway, North East ; Franklin Burr, Lindley ; Morris W. Cowden, Gerry ; Thomas G. Corlett, Buffalo ; Abraham F. Crans, Owego ; Wm. H. Crowley, Rainbow ; Chas. Edson Davis, Mecklenburg ; M. Emma Dickinson, Geneseo ; Elmer E. Eddy, La Fargeville ; Robert M. Elliott, Gasport ; Wm. F. Elmendorf, Buffalo ; Albert F. Erb, A. M., Clarence ; Richmond E. Gibson, Coudersport ; Thomas H. Hallett, Huron ; Herman Hellenstein, New York City ; Jacob E. Helwig, Clarence ; Harrie B. Howell, Rochester ; Wm. J. Humphrey, Union City ; Wm. L. Hunter, Lockport ; Burt C. Johnson, Gowanda ; Thomas R. Jones, A. B., Ripon ; Wm. E. Kay, Otterville ; Chetwode H. W. King, Buffalo ; Heinrich C. Leonhardt, Logan ; Charles T. Loritz, Rochester ; Thomas B. Loughlen, Olean ; Frederick E. McClellan, Canandaigua ; John J. McCullough, Buffalo ; Charles S. Meahl, Clarence Centre ; Jerry C. Murphy, Humphrey ; Arthur T. O'Hara, Albion ; Chas. B. Osborne, Burdette ; Jeanette M. Potter, Ithaca ; Alice R. MacLean Ross, Buffalo ; Marie S. Ross, Buffalo ; Addison W. Scott, Fayette : Jacob J. Simonds, Shelby ; Chas. S. Smith, Bath ; Franklin A. Stevens, Sinclairville ; Walter Stuart, Harmony ; Clara B. Talbot, Rockport ; Wm. Teft, Hornellsville ; Daniel J. Tillotson, Victor ; George W. Tong, Tonawanda ; Frederick B. Willard, Geneseo ; John White, Williamsport ; James Franklin Whitwell, Medina.

The honor roll was announced as follows :—Messrs. King, White, Miss Bender, Johnson, Tong, Brockway, Miss Alice Ross, Davis, Humphrey, Burr and Willard.

Mr. Willard received honorable mention for the general excellence and originality of his thesis.

The degree of Graduate in Pharmacy was conferred upon the following class :

John McCullough Bargar, Sinclairville, N. Y. ; Lucius Rinaldo Blackney, Buffalo ; Herman Cornelius Cleveland, Buffalo ; Elizabeth

Dort, Buffalo; Mathias Joseph Frisch, Buffalo; Charles Henry Gauger, Rochester; Frank Hall Goler, Rochester; William Charles Heussy, Buffalo; William George Holser, Buffalo; John Christian Krieger, Salamanca, N. Y.; Frank Gardner Prescott, Buffalo; George Benjamin Rogers, Buffalo; Eugene Ariel Spenzer, Cleveland; Edward Everett Stanbro, Springville, N. Y.; Arthur Lee Williams, Clayton, N. Y.; William August Ziemendorf, St. Joseph, Mo.

The Rev. Eliphalet Nott. Potter, D. D., LL. D., President of Hobart College, gave the address to the graduating class. The address to the Alumni was given by the Rt. Rev. A. Cleveland Coxe, D. D., LL. D.

After the exercises, the annual banquet was held at the Tifft House.

The Spring term will begin Monday, March 31st, and continue eight weeks. All correspondence relating to the Spring course should be addressed to Dr. DeLancey Rochester, Secretary, 469 Franklin street.

